# Patterns and processes of somatic mutations in nine major cancers

**DOI:** 10.1186/1755-8794-7-11

**Published:** 2014-02-19

**Authors:** Peilin Jia, William Pao, Zhongming Zhao

**Affiliations:** 1 Department of Biomedical Informatics, Vanderbilt University School of Medicine, Nashville, TN 37203, USA; 2 Center for Quantitative Sciences, Vanderbilt University Medical Center, Nashville, TN 37232, USA; 3 Department of Medicine, Vanderbilt University School of Medicine, Nashville, TN 37232, USA; 4 Department of Cancer Biology, Vanderbilt University School of Medicine, Nashville, TN 37232, USA; 5 Vanderbilt-Ingram Cancer Center, Vanderbilt University Medical Center, Nashville, TN 37232, USA

**Keywords:** Somatic mutation, Cancer, *Kataegis*, Mutation signature, Mutagen, Heterogeneity

## Abstract

**Background:**

Cancer genomes harbor hundreds to thousands of somatic nonsynonymous mutations. DNA damage and deficiency of DNA repair systems are two major forces to cause somatic mutations, marking cancer genomes with specific somatic mutation patterns. Recently, several pan-cancer genome studies revealed more than 20 mutation signatures across multiple cancer types. However, detailed cancer-type specific mutation signatures and their different features within (intra-) and between (inter-) cancer types remain largely unexplored.

**Methods:**

We employed a matrix decomposition algorithm, namely Non-negative Matrix Factorization, to survey the somatic mutations in nine major human cancers, involving a total of ~2100 genomes.

**Results:**

Our results revealed 3-5 independent mutational signatures in each cancer, implying that a range of 3-5 predominant mutational processes likely underlie each cancer genome. Both mutagen exposure (tobacco and sun) and changes in DNA repair systems (APOBEC family, *POLE*, and *MLH1*) were found as mutagenesis forces, each of which marks the genome with an evident mutational signature. We studied the features of several signatures and their combinatory patterns within and across cancers. On one hand, we found each signature may influence a cancer genome with different influential magnitudes even in the same cancer type and the signature-specific load reflects intra-cancer heterogeneity (e.g., the smoking-related signature in lung cancer smokers and never smokers). On the other hand, inter-cancer heterogeneity is characterized by combinatory patterns of mutational signatures, where no cancers share the same signature profile, even between two lung cancer subtypes (lung adenocarcinoma and squamous cell lung cancer).

**Conclusions:**

Our work provides a detailed overview of the mutational characteristics in each of nine major cancers and highlights that the mutational signature profile is representative of each cancer.

## Background

Somatic mutations are a major cause of cancer development [1]. Recent advances in next-generation sequencing (NGS) technologies have revealed that hundreds to thousands of somatic nonsynonymous mutations could exist in a single cancer genome [2-7]. The causes and forces that lead to these mutations remain largely unknown. In cancer research, somatic mutations can be categorized as driver mutations and passenger mutations. Driver mutations are defined as those that confer growth advantages to tumor cells and are under positive selection [8]. In contrast, passenger mutations do not contribute to cancer development [8]. While selection has been well considered as a mutational force that operates within cancer genomes, a previous study has shown that selection affects only a small amount of the mutations and the mutation patterns are not significantly biased by either positive or negative selection [9].

Recently, several pan-cancer genome studies have been reported by The Cancer Genome Atlas (TCGA), aiming to study simultaneously thousands of cancer genomes across many cancer types [10-14]. Through these studies, a total of 21 mutational signatures were extracted from 30 types of cancers, providing not only the landscape but also a dictionary of mutational signatures in major cancers [10]. These signatures not only confirmed previously recognized internal and external risk factors involving DNA damage, such as environmental DNA-damaging agents, tobacco carcinogens [15,16], radiation, and chemicals, but also revealed novel mechanisms that mark cancer genomes with specific mutational patterns [2,17]. For example, the *kataegis* signature was found to be highly correlated with a DNA repair process mediated by the AID/APOBEC family proteins [18]. In addition to mutagen-driven forces (e.g., tobacco smoking and sun exposure), failures in DNA repair systems lead to a rapid accumulation of somatic mutations in cancer genomes. In fact, somatic mutations in each cancer genome could reflect the lifetime interplay between DNA damage and DNA repair processes in cancer patients [17,19]. To date, the mechanisms discovered that could disrupt DNA repair genes include somatic mutations [20], copy number alterations [21], dysregulated gene expression [22], and epigenetic changes [23]. For example, hypermethylation in the *MLH1* gene, and correspondingly, its decreased expression level, could result in microsatellite instability (MSI) [24]. In summary, heterogeneous mutational signatures and processes were highlighted as a prevalent phenomenon in cancer [13], further complicating the studies of cancer somatic mutations.

While these pioneering studies have established the first architecture of somatic mutation patterns in pan-cancers, a detailed and deep exploration of mutational profiles within and between cancer types has not yet been performed. For example, how many heterogeneous mutation forces directly affect each cancer type and each single cancer genome? And, to what extent does each process act to modulate and shape the mutational spectrum we have observed in cancer genomes? Furthermore, while mutagen exposures, such as tobacco smoking, drive specific mutation patterns that are detectable, it has not yet been established whether DNA repair systems behave similarly on cancer genomes, e.g., form specific mutation patterns.

In this work, we implemented the Non-negative Matrix Factorization (NMF) algorithm [18] to decompose and detect somatic mutation signatures in nine major cancers. Although some signatures have been previously reported, our analysis reveals heretofore unrecognized features. In contrast to pan-cancer signatures, we aim to study the signatures in each cancer and to investigate intra- and inter-cancer mutation signature profiles. First, our results revealed 3-5 independent mutational signatures in each cancer, implying that 3-5 primary mutational processes are critical for tumorigenesis. Specifically, we identified three mutagen-driven signatures, three DNA-repair related signatures, and one recurrent signature with C → T mutations at non-CpG island (CGI) regions. Second, our assessment of the mutagen-driven mutational loads (e.g., the smoking related signature in smokers versus never-smokers) in tumor genomes revealed correlations with the clinical data and demonstrated intra-cancer heterogeneities of the same cancer type. Additionally, we observed different combinatory patterns of mutation signatures in each cancer type, highlighting the prevalent heterogeneities among different cancers. These results provide a detailed overview of the mutational signatures in each of the nine major cancers.

## Methods

### Cancer mutation data

As summarized in Table 1, we collected somatic single nucleotide variants (SNVs) in nine major cancers from several large-scale NGS projects: breast cancer (BrCa_21 with data from reference [18] and 507 TCGA BRCA [25]), 224 colon and rectal cancers (CRC) [26], 248 endometrial carcinomas (EC) [27], 290 glioblastoma (GBM) [23], 74 head and neck squamous cell carcinomas (HNSCC) [6], 182 lung adenocarcinomas (LUAD) [15], 121 melanoma [28], 316 ovarian carcinomas (OvCa) [29], and 177 squamous cell lung cancers (SQCC) [30]. Six of these cancer datasets were from TCGA: BRCA, CRC, EC, GBM, OvCa, and SQCC. For these, the glioblastoma mutation data was downloaded recently (May 12, 2013) and the other five cancers were downloaded using the data reported in the original publications (Table 1). With the exception of the 21 breast cancer samples whose data was generated through whole-genome sequencing (WGS), all the data in the remaining samples was based on whole exome sequencing (WES). The downloaded mutation data was all previously mapped to gene regions. Therefore, among WES samples, the scale of the mutation numbers per sample is comparable to each other.

**Table 1:** Description of the mutation datasets

**Dataset**	**Cancer type**	**Sequencing platform**	**# samples**	**# SNVs**	**Pub. year**	**Reference**
HNSCC	Head and neck squamous cell carcinoma	WES	74	9398	2011	Stransky et al. [6]
LUAD	Lung adenocarcinoma	WES, WGS	182	62,767	2012	Imielinski et al. [15]
Melanoma	Melanoma	WES	121	220,430	2012	Hodis et al. [28]
TCGA_BRCA	Breast tumors	WES	507	31,538	2012	TCGA [25]
TCGA_CRC	Colon and rectal cancer	WES	224	90,059	2012	TCGA [26]
TCGA_EC	Endometrial carcinoma	WES	248	181,815	2013	TCGA [27]
TCGA_GBM	Glioblastoma	WES	290	20,949	2008	TCGA [23]
TCGA_OvCa	Ovarian carcinoma	WES	316	18,296	2011	TCGA [29]
TCGA_SQCC	Squamous cell lung cancer	WES	177	64,339	2012	TCGA [30]

SNVs: single nucleotide variants.

We download the Consensus CDS (CCDS) information and the respective coding DNA sequences, CpG island data, and human reference genome (hg18 and hg19) from the UCSC Browser [31]. Gene expression data was retrieved from the TCGA data portal [32]. The *POLE* mutation data for CRC samples was downloaded from the cBio Portal for Cancer Genomics [33]. The *POLE* mutation data for EC was extracted using the “Integrative Cluster” information provided by the original study [27]. The MSI status data for both CRC [26] and EC [27] were downloaded from the original work.

### Mutation signature detection

Non-negative Matrix Factorization (NMF) has been frequently applied in detecting mutation signatures from somatic mutation data. A detailed description of NMF can be found in a previous study [18] and in Additional file 1: Text S1. Briefly, given a mutation matrix **M** in which rows represent the 96 available trinucleotides and columns represent tumor samples, NMF factorizes **M** into two matrices, **W** and **H**, i.e., **M**_96×*N*_ = **W**_96×*r*_×**H**_*r*×*N*_ + ϵ, where *r* is the factorization rank corresponding to the number of mutational signatures to be detected, and *N* is the total number of samples. *r* is determined by evaluating the cophenetic correlation and sparseness. The matrix **W**_96×*r*_ contains *r* columns, each of which represents a mutational signature. We assume that an observed signature in a cancer genome is produced by a major mutational process. The matrix **H**_*r*×*N*_ contains *N* columns, where H_*r*×*j*_ = [β_*i,j*_]^T^, *i* = 1:*r* and *j* = 1:*N*. The coefficient β_*i,j*_ represents the load of the *i*^th^ signature on the fraction of mutations in the *j*^th^ sample.

## Results

### Each cancer has distinct and distinguishable mutational signatures

Although pan-cancer analyses have been comprehensively performed in recent studies, cancer type specific mutation signatures were only explicitly examined in breast cancer. Nik-Zainal et al. [18] and Fischer et al. [34] proposed that breast cancer genomes had 5 biologically distinct signatures. One of these signatures is called *kataegis* and is a phenomenon of regional hyper-mutation in cancer genomes. We first replicated the results of this earlier work using the same WGS dataset of 21 samples (denoted as BrCa_21). With this proof of the concept, we then applied the approach to each of the nine cancers, including a WES dataset of 507 TCGA breast cancer samples (Table 1). The overall results and performance assessment in each cancer using different *r* are provided in Additional file 2: Table S1.

**Figure 1:**
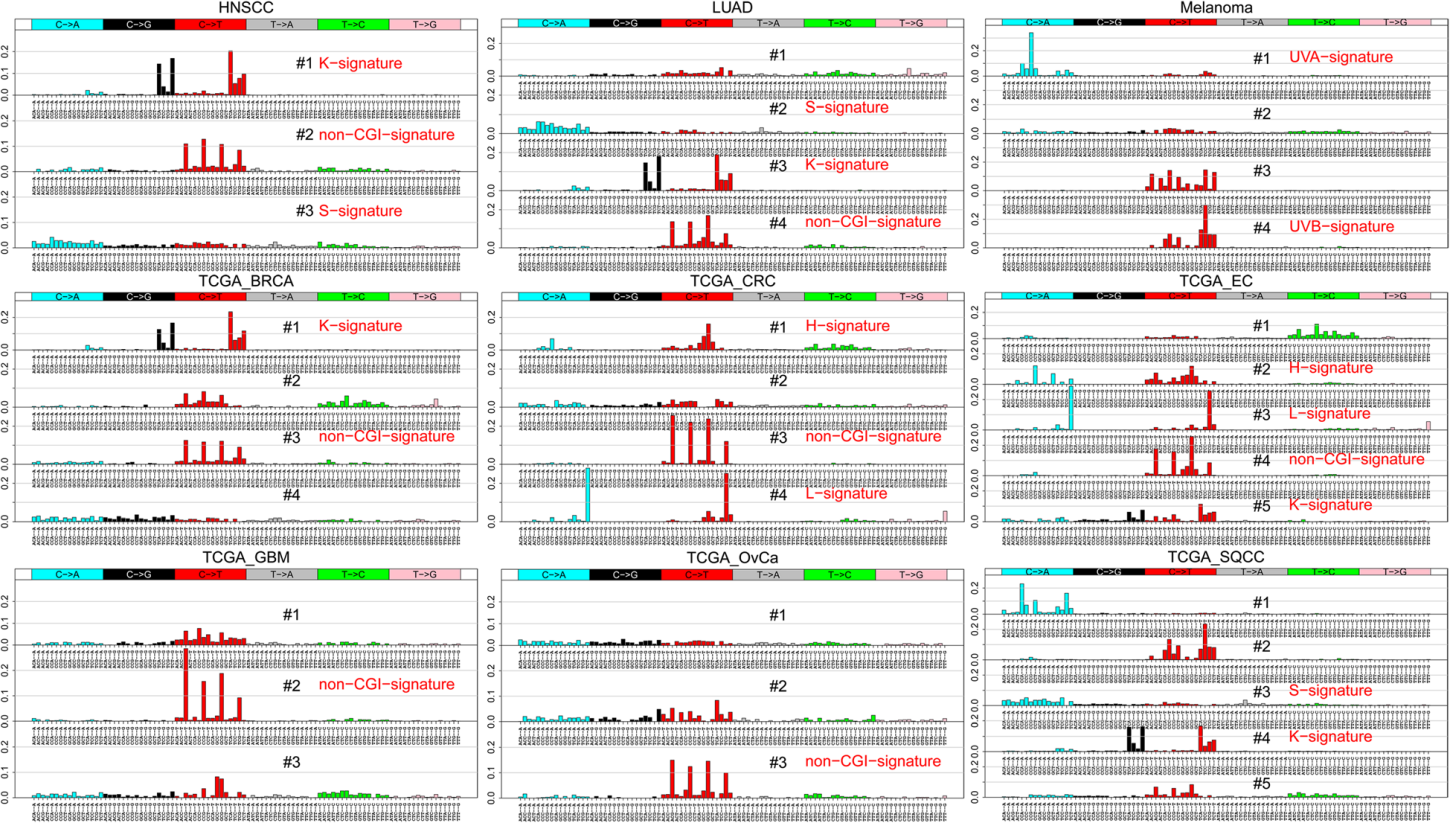
**Mutational signatures in 9 types of cancer.** X-axis denotes the 96 trinucleotide substitutions. Y-axis denotes the relative coefficient of each substitution to the corresponding signature (the matrix **W**_96×*r*_, see the main text). For each cancer, the plot in each panel represents one detected signature. HNSCC: head and neck squamous cell carcinoma. LUAD: lung adenocarcinoma. TCGA_BRCA: the breast cancer data from TCGA. TCGA_CRC: colon and rectal cancer. TCGA_EC: endometrial carcinoma. TCGA_GBM: glioblastoma. TCGA_OvCa: ovarian carcinoma. TCGA_SQCC: squamous cell lung cancer.

In previous pan-cancer studies, Alexandrov et al. [10] pooled all 30 cancers and identified 21 signatures. Here, we focused on the signature profile in each cancer type and applied NMF independently in each cancer. For better comparison, we used the same color scheme and plot style as in the original work [18] and in Alexandrov et al. [10]. As shown in Figure 1, the NMF method achieved the best fitting of the mutation matrices with four signatures in most of the nine cancers, with a narrow range of three to five. HNSCC, GBM, and OvCa were found to have three signatures each. EC and SQCC had five signatures each. All the remaining cancers had four signatures. Thus, complex but limited patterns of mutational signatures are detectable in most cancer genomes. Assuming each signature is primarily driven by a major process, somatic mutations would be the outcome of multiple independent biological processes in cancer genomes. No single process can explain all somatic mutations in any cancer. Furthermore, while several signatures are recurrent in multiple cancers (see below), inter-cancer differences are remarkable in their combination of multiple signatures for each cancer type. Table 2 summarizes the mutational signature profile for each cancer.

**Table 2:** Summary of mutational signatures in 9 cancers

**Dataset**	**# signatures**	**Signature**				
		**Smoking related (S-signature)**	**UV-related signatures**	**K-signature (C → T and C → G in TCX)**	**H- and L-signature**	**Non-CGI signature (C → T in XCG)**
HNSCC	3	√		√		√
LUAD	4	√		√		√
Melanoma	4		√			
TCGA_BRCA	4			√		√
TCGA_CRC	4				√	√
TCGA_EC	5			√	√	√
TCGA_GBM	3					√
TCGA_OvCa	3					√
TCGA_SQCC	5	√		√		

Details of datasets are provided in Table 1. CGI: CpG island.

### Features of representative signatures

#### The S-signature: smoking-related signature (mutagen-driven)

Tobacco and sun exposure are well-known mutagen sources that cause DNA damage and have specific mutation patterns. The fingerprint mutation due to tobacco exposure is a C → A transversion, which is predominantly found in smokers [15]. In both LUAD and SQCC, the C → A mutations appeared as an independent signature (LUAD signature #2 and TCGA_SQCC signature #3, the cyan bars, Figure 1). We denoted it as the S-signature, reflecting the effect of smoking. Notably, the S-signature has moderate coefficients for all 16 trinucleotides related to C → A, implying that among the tobacco-driven C → A mutations, there is no particularly favorable neighboring sequence context. It is worth noting that, in HNSCC, we also observed the S-signature (HNSCC signature #3, Figure 1), consistent with the observation that many HNSCC patients have a history of tobacco smoking.

#### The UVA- and UVB-signatures: sun-exposure related signatures (mutagen-driven)

In nature, sun exposure leads to DNA damage through three major types of UV light. UVC is largely blocked by the ozone layer, while UVA and UVB in strong sun light are the main sources of UV light-produced DNA damage. UVA primarily induces C → A [28,35], and UVB induces C → T [28] mutations; however, recent studies found that UVA might lead to C → T mutations as well [36,37]. In melanoma, the first signature is indicative of UVA exposure, as it features C → A mutations (melanoma #1, the cyan bars, Figure 1, denoted as UVA-signature), the fourth signature features C → T (melanoma #4, the red bars, Figure 1), likely due to UVB contribution (denoted as UVB-signature). DNA damage due to UV exposure leads to covalent bonds between two adjacent pyrimidines (Py) [38]; therefore, mutations at Cs created by UV light usually occur in the context of bipyrimidines [19]. In our results, both UVA- and UVB-signatures (melanoma #1 and #4) favored pyrimidines at the 5’ side of the mutation site. For example, **C**(C → A)X in the UVA-signature and **C**(C → T)X and **T**(C → T)X in the UVB-signatures had relatively high coefficients. This result not only supported that those signatures were UV related, but also confirmed a prevailing Py-C mutation pattern led by UV exposure.

#### The K-(kataegis) signature: C → T and C → G in a trinucleotide, TCX

In the previous investigation by Nik-Zainal et al. [18], C → T mutations in the TpCpX trinucleotide context clustered within genomic regions of several megabases (i.e., in *cis*-fashion) and tended to occur with a special strand preference. For example, the C → T mutations occur continuously on one strand and then jump to the reverse strand (G → A); however, they do not mix on the same strand. The transversion of C → G enriched at the TpC dinucleotide context has also been observed previously in lung and ovarian cancers [2]. In our work, we confirmed the *kataegis* signature (abbreviated as the K-signature in Figure 1) in breast cancer (TCGA_BRCA signature #1, Figure 1), endometrial carcinoma (TCGA_EC #5), head and neck squamous cell carcinoma (HNSCC #1), lung adenocarcinoma (LUAD #3), and squamous cell lung cancer (TCGA_SQCC #4), featured with C → T and C → G mutations in the context of the TCX trinucleotide. The C → T mutations in the TpC dinucleotide context related to the *kataegis* signature have been associated with the AID/APOBEC mediated DNA repair system [18,22,39,40]. We also systematically examined all 11 members of the APOBEC family using the TCGA RNA sequencing (RNA-seq) data (Additional file 3: Table S2 and Additional file 4: Table S3). Positive correlations were observed between the K-signature related mutation burden and increased expression of *APOBEC3B* or *APOBEC3A* (see Additional file 5: Figure S1), which is consistent with previous reports [40].

#### The L-(low level MSI or MSS) signature: T(C → A)T and T(C → T)G, and the H-(high level MSI) signature: C(C → A)X and G(C → T)X

**Figure 2:**
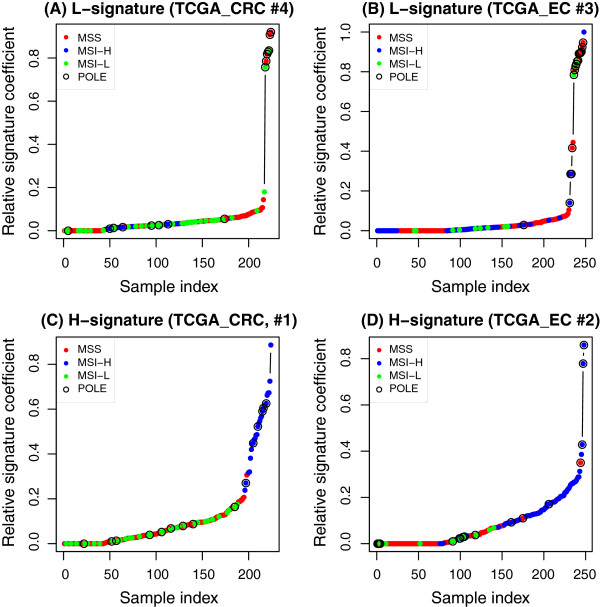
**Distribution of the L- and H-signatures in colon and rectal cancer (TCGA_CRC) and endometrial carcinoma (TCGA_EC).** X-axis: sample indexes. Y-axis: relative coefficient of the signature. In the L-signature, samples with a microsatellite stable status (MSS, red dots) or low levels of a microsatellite instable status (MSI-L, green dots) have high coefficients in both colon and rectal cancer **(A)** and endometrial carcinoma samples **(B)**. In the H-signature, samples with high levels of MSI (MSI-H, blue dots) have high coefficient in both colon and rectal cancer **(C)** and endometrial carcinoma samples **(D)**. Samples with *POLE* mutations were denoted by large circles.

In colon and rectal cancer and endometrial carcinoma, we observed two shared signatures. The first signature features T(C → A)T and T(C → T)G (TCGA_CRC #4 and TCGA_EC #3, Figure 1). This signature was found with high coefficients (i.e., more importance) in samples with low levels of a microsatellite instable (MSI-L) status (green dots in Figure 2A) or a microsatellite-stable (MSS) status (red dots in Figure 2A) in both CRC and EC samples. We thus denoted it as the L-signature for low-level MSI or MSS status. The second signature contains C(C → A)X and G(C → T)X (TCGA_CRC #1 and TCGA_EC #2, Figure 1) and was found to play more roles in samples with mutant *POLE* and high levels of MSI status (MSI-H) (Figure 2C and D, blue dots). We denote this signature as the H-signature for high-level MSI status. These results indicated that the L- and H-signatures were likely associated with the microsatellite status and deficiencies in the DNA repair gene *POLE*.

**Figure 3:**
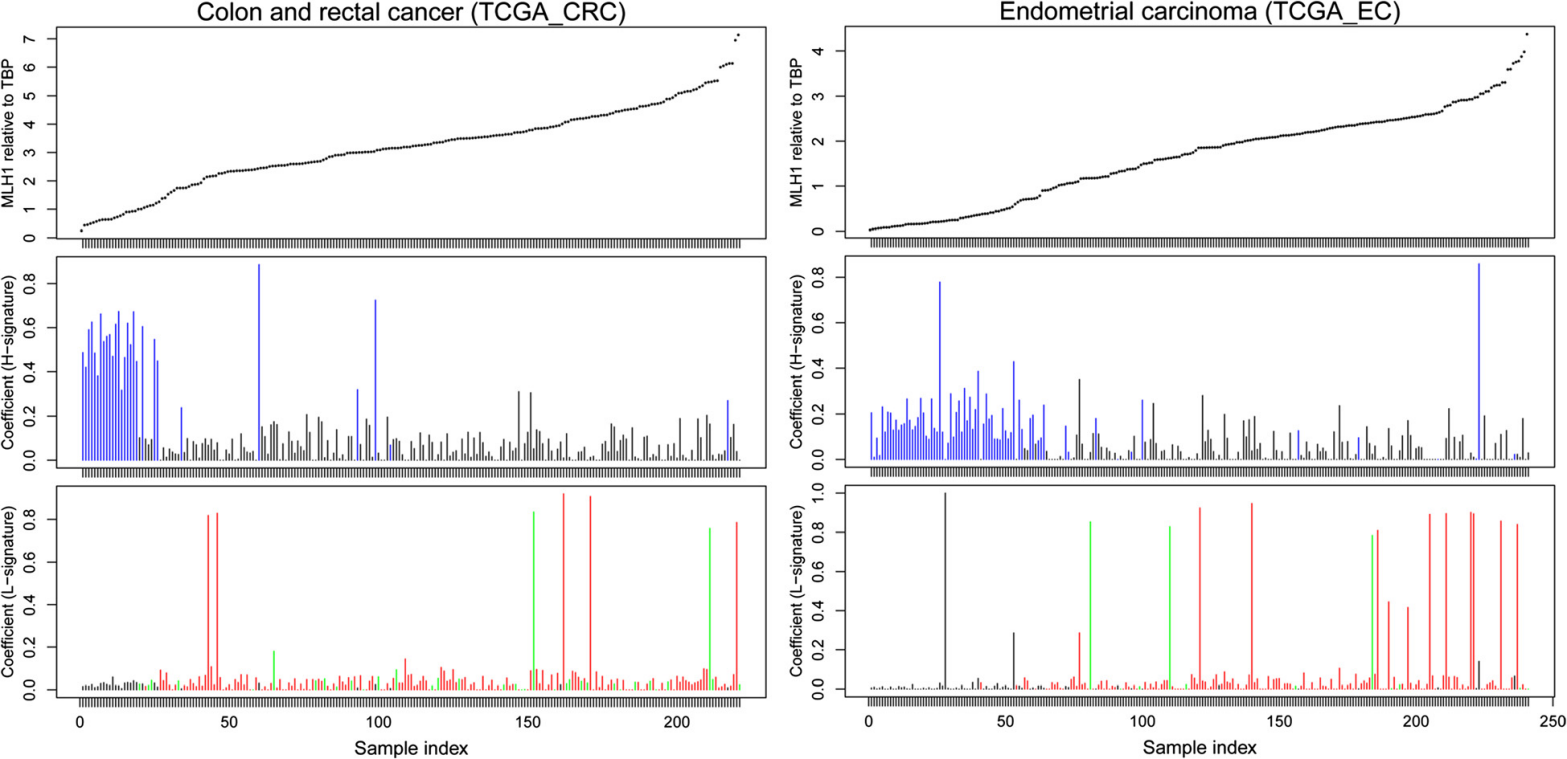
**Coefficient distribution of the L- and H-signatures in samples ordered by *MLH1 *gene expression level.** Top panel: *MLH1* gene expression relative to the house-keeping gene, *TBP*, sorted. Middle panel: the H-signature coefficients in each sample. Samples are listed in the same order as on the top panel. Samples with a high level of MSI are labeled in blue. Bottom panel: the L-signature coefficients in each sample. Samples are in the same order as on the top panel. The figure shows that the samples with a high coefficient of signature #1 had low expression values of *MLH1* gene.

Considering the relationship between L- or H-signatures and microsatellite status, we explored their correlations in the context of the *MLH1* expression level, which was reported to cause microsatellite instability. As expected, CRC samples with high relative H-signature coefficients tended to distribute both toward a low *MLH1* expression and have a MSI-H status (blue vertical lines in Figure 3). In contrast, the seven CRC samples with the highest coefficients had no obvious correlation with *MLH1* expression levels. A similar trend was observed in endometrial carcinoma samples: samples with low *MLH1* expression tended to have high H-signature coefficients. For the L-signature, EC samples with high coefficients seemed to be randomly distributed in samples with low *MLH1* expression levels. In summary, the H-signature with C(C → A)X and G(C → T)X tended to occur in MSI-H samples with a low expression of *MLH1,* while the L-signature with T(C → A)T and T(C → A)G occurred mainly in MSS or MSI-L samples and had no apparent link with *MLH1* gene expression.

#### The non-CGI-signature: C → T in CG dinucleotides in non-CpG island (CGI) regions

We found a signature characterized by C → T in the context of CG dinucleotides in seven of the nine cancers: BRCA, CRC, EC, GBM, HNSCC, LUAD, and OvCa. The signature was not detected in melanoma. The mutation patterns in this signature resemble the age-related signatures discovered in Alexandrov et al. [10] (called “signatures 1A/1B”).

The mutation rate at nucleotide C in the methylated CpG dinucleotide context has been found at 10- to 50-fold higher levels than that of other sites [41]. CpG dinucleotides often cluster in the genome and form CGIs, even though they occur at only approximately 25% of the expected frequency in the human genome [42]. To explore the correlation between this signature and the features of CGIs, we applied NMF to specifically examine X(C → T)G mutations that were located inside and outside of CGIs, respectively, in the seven cancers in which the signature was detected. As shown in Additional file 6: Figure S2, by comparing the results obtained with or without X(C → T)G mutations in non-CGI regions, we found that the signature was substantially diminished, or even barely detectable, after excluding X(C → T)G mutations in non-CGI regions in all seven cancers. This finding indicated that this signature was mainly formed by X(C → T)G mutations in non-CGI regions. Accordingly, we termed it the non-CGI-signature.

### Intra-cancer heterogeneity: mutational loads are concordant with and indicative of clinical data

**Figure 4:**
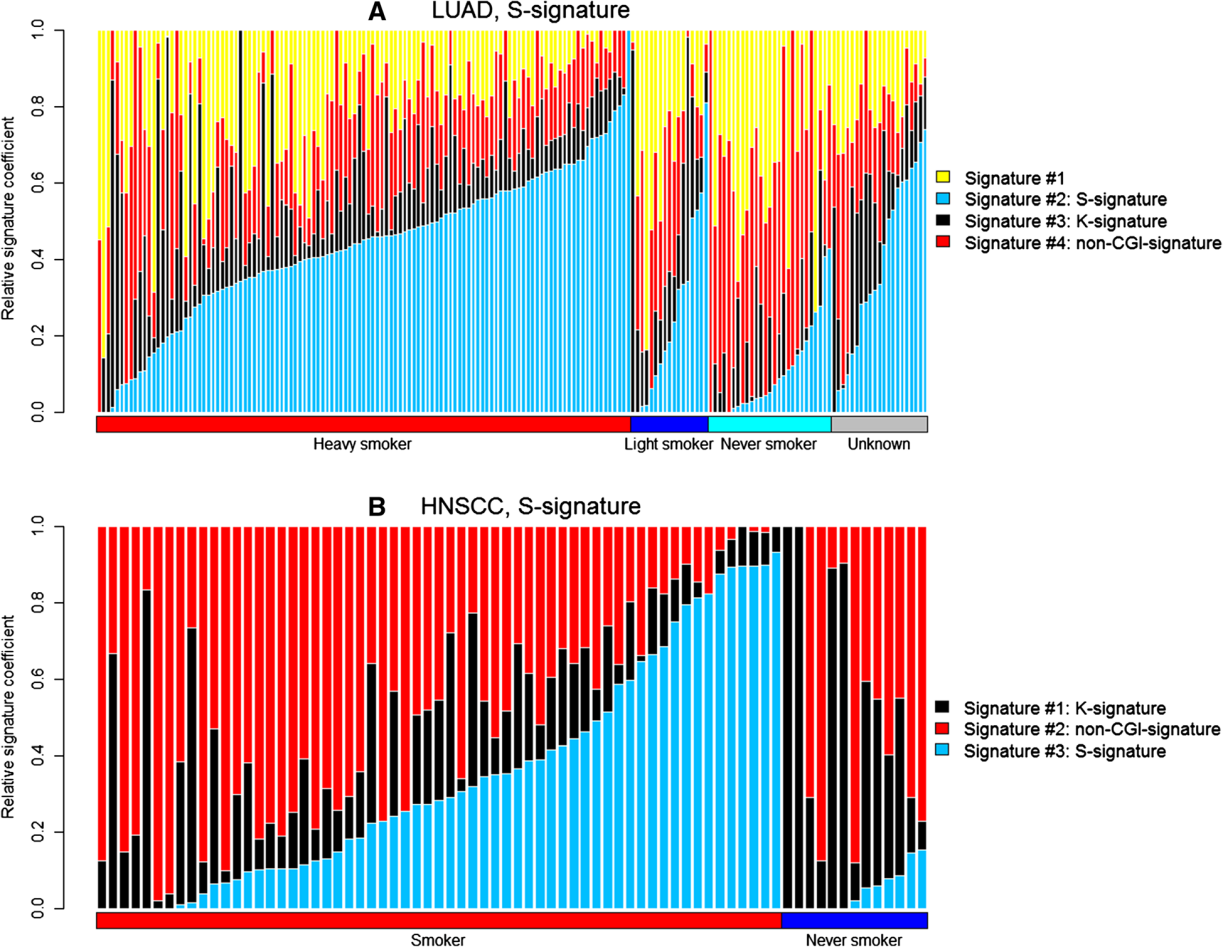
**Contribution of the S-signature (represented by the vertical blue bars) to each lung adenocarcinoma (LUAD) sample (A) and to the head and neck squamous cell carcinoma (HNSCC) sample (B), measured by the relative coefficients.** The relative coefficients were computed based on the true coefficients such that the sum of the relative coefficients for all signatures in each sample equals to 1. Blue: the S-signature. Black: the K-signature. Red: the non-CGI-signature. Yellow: the random signature.

We explored the biological and clinical implications of the observed mutational signatures. We hypothesized that if a signature were indeed generated by a mutagen exposure process, the mutation load that it forms as a fraction of the overall mutation load of the sample would be correlated with the patient’s smoking or sun exposure history. Here, the signature-specific mutation load is measured by its relative coefficient, calculated by the actual coefficient divided by the sum of all coefficients in each sample such that the sum of all relative coefficients per sample equals 1. Among the four cancers with mutagen-driven signatures (HNSCC, LUAD, melanoma, and SQCC, Table 2), the LUAD and HNSCC datasets provide smoking exposure information. The LUAD samples were categorized into four groups according to the number of consumed packs of cigarette per year: heavy smoker, light smoker, never smoker, and unknown [15]. By overlaying the smoking status to the distribution of the signature mutation load in each sample, we found the S-signature contributed to heavy, light, and never smokers with a steadily decreasing scale (Figure 4A). A similar trend was observed in HNSCC samples (Figure 4B). Based on the clinical data, we denoted HNSCC never smokers as those that consume zero packs of cigarettes per year; all other samples were smokers [6]. The relative coefficients of the S-signature (HNSCC #3) were significantly lower in HNSCC never smokers compared to HNSCC smokers (*p* = 9.49 × 10^-6^, t-test). Furthermore, tumors with heavy loads of the S-signature (i.e., those with the S-signature coefficient ranked between 1-50% in a decreasing order) were associated with significantly more cigarette packs per year than those with light loads (ranked between 51-100%) (*p* = 0.003, t-test).

In melanoma, where certain types arise from sun-exposed or non-sun-exposed areas, we performed a similar analysis to investigate the correlation between UV-related signatures and the anatomical origins of different tumors. As shown in Additional file 7: Figure S3, acral melanomas (that arise on the hands and feet) tend to have a smaller load of UVA-/UVB-signatures compared to those from body skin. These observations collectively indicate that the mutational signatures we observed provide implications for their biological mechanisms and clinical histories. In addition, patients with the same cancer type, and even the same subtype (e.g., smokers), have different load of each signature, implying intra-cancer differences being prevalent.

### Inter-cancer combinatory patterns of mutational signatures are distinguishable between cancer types

Based on the mutational signatures and their potential biological implications above, we next examined the combinatorial patterns of signatures for each cancer. As shown in Table 2, no cancer types share the same combination of signatures (i.e., signature profile), even though several signatures are present in two or multiple cancers. For example, three cancers have both mutagen-driven and DNA repair related processes: HNSCC, LUAD, and SQCC, all of which have the smoking related S-signature and the APOBEC related K-signature. Of note, even the two types of lung cancer, LUAD and SQCC, have distinct mutation patterns. LUAD has the age-related non-CGI-signature but SQCC does not, further demonstrating the extensive heterogeneous mutations and processes underlying each cancer. A similar pattern was also observed in ref. [10], where lung SQCC samples were found to have 3 signatures (APOBEC, smoking, and signature 5 in [10]) plus an unknown signature named “other”, lung small cell samples were found with 2 (smoking and signature 15 in [10]), and LUAD were found with 4 (age, APOBEC, smoking, and signature 5 in [10]) plus the “other” signature. Three cancers have only DNA repair related processes: BRCA, CRC, and EC, none of which have an explicit mutagen-driven related process. One cancer, melanoma, has only mutagen-driven signatures: the UVA- and UVB-signatures. The remaining two cancers, GBM and OvCa, do not have signatures confidently linking to either a mutagen-driven or DNA repair related process. Even though some cancers have private signatures (i.e., not observed in any other cancers), such as TCGA_GBM #1 and TCGA_SQCC #1 (Figure 1), it is actually not the private signatures but rather the combination of mutation patterns that distinguish these cancers. In summary, both common and unique signatures exist among the nine cancers that we examined, yet the combinations of mutational signatures are representative and distinct in each cancer.

## Discussion

We systematically analyzed mutational signatures among nine major types of cancer, each of which displayed various extents of heterogeneity in mutational mechanisms. Our results revealed that the number of mutational processes within each type of cancer is in a small range, i.e., 3-5, with no single process being qualified to explain the overall somatic mutations in any individual cancer type. Both intra-cancer and inter-cancer heterogeneity is well recognized and represented in the mutational signatures we identified. In cancers with known or traceable mutagenic sources, such as lung cancers and melanoma, signatures contributed by DNA damage seem to be the major mutation processes. On the contrary, several other cancers, such as BRCA, CRC, and EC, have mutational spectra that are shaped by DNA repair systems, such as somatic mutations in DNA repair genes or abnormal gene expression.

Although many of these signatures were reported right after we completed our analyses, our work focused on the mutational signatures within each single cancer type and studied in more detail each signature, providing the following new insights into cancer research. First, while heterogeneity has been highly appreciated in recent investigations, only 3-5 independent signatures were found in each of the nine cancers we examined, implying that a small number of primary mutational processes could mainly shape a cancer genome. This is an estimation based on the current data, and in a recent pan-cancer analysis [10], the number of processes was reported to be between 2 and 6 in each of the 30 cancers. There are several reasons for the differences. The previous work did not count the “other” signature. For example, both GBM and OvCa were denoted to have three signatures in the present work but two signatures plus the “other” signature in Alexandrov et al. [10]. Sample difference is another major reason for the differences between our work and the previous work. Most of the cancers harboring 6 signatures are not included in our work. Outlier samples may also lead to differences in mutation signatures. For example, we observed the UVB-signature in SQCC unexpectedly. However, by exploring the load of each signature in each sample, one SQCC sample contributed predominantly to the UVB-signature of SQCC (Additional file 8: Figure S4). After excluding this outlier sample, four major mutation signatures were identified for SQCC, indicating that the UVB-signature we observed is not a prevalent signature in SQCC.

Second, the mutation load enforced by each signature and its underlying process are detectable. In particular, the mutation load resulting from mutagen related signatures, as linked to the mutagen driven processes, is indicative of exposure history and clinical data. For example, based on the mutation load of the S-signature, the LUAD samples shown in Figure 4 with an unknown smoking status are implied to be heavy or light smokers rather than never smokers.

Third, both intra- and inter-cancer differences are indicative from the mutation signature profiles. These differences are well-represented by the mutation load in each single cancer samples with the same cancer type or subtype, as well as, by the combinatorial pattern of multiple mutation signatures in each cancer type.

This work has the following limitations. First, there are still several signatures whose biological mechanisms remain unclear, including several private signatures that were uniquely observed in only one specific type of cancer. For example, the first signature in EC (TCGA_EC #1) features T → C mutations and the first signature in SQCC (TCGA_SQCC #1) has several C → A mutations (Figure 1). Future work is needed to interpret these signatures, perhaps with the incorporation of high-throughput genetic and genomic data from multiple domains as well as whole genome sequencing data. Second, the number of mutational processes selected for each cancer was determined by the cophenetic correlation and sparseness calculations but may not always be the best fit within biological systems. For most cancers, a single best model can be accomplished upon the detection of the number of applicable mutational processes. However, a manual check is necessary for performance optimization. For example, a previous study discovered 5 mutational signatures in 21 breast cancer samples [18]. In our work, we found 4 signatures using TCGA_BRCA samples. Our further inspection of those models with 4 signatures and 5 signatures concluded that 4 is appropriate for the TCGA_BRCA data because when using 5 signatures, the fifth signature stood out as a duplicate random mutational process.

## Conclusions

In this study, we implemented the NMF method to dissect the mutational profiles of nine major cancers. Our results revealed 3-5 independent mutational signatures in each cancer, implying heterogeneous mutational processes prevalently exist in cancer genomes to modulate the somatic mutational spectra. Both mutagen exposure (e.g., tobacco and sun) and changes in DNA repair systems are capable of producing DNA damage that results in major mutational signatures in cancer genomes. We revealed features for several important signatures. Samples of the same cancer type were found with markedly different load of the same signatures, e.g., the S-signature in smokers versus never smokers, indicating a prevalent heterogeneity within (intra-) cancer types. In contrast, samples of different cancer types showed different profiles of mutational signatures, highlighting the heterogeneity among (inter-) different cancer types. This work provides a unique overview of somatic mutations in each of the nine cancers.

## Abbreviations

BRCA: Breast cancer; CCDS: Consensus coding DNA sequence; CGI: CpG island; CRC: Colon and rectal cancer; EC: Endometrial carcinoma; GBM: Glioblastoma; HNSCC: Head and neck squamous cell carcinoma; LUAD: Lung adenocarcinoma; MSI: Microsatellite instable (or instability); MSS: Microsatellite stable (or stability); NGS: Next-generation sequencing; NMF: Non-negative matrix factorization; OvCa: Ovarian carcinoma; SQCC: Lung squamous cell carcinoma; WES: Whole exome sequencing; WGS: Whole-genome sequencing.

## Competing interests

The authors declare that they have no competing interests.

## Authors’ contributions

PJ and ZZ conceived and designed the study. PJ collected the data and conducted data analysis. PJ, ZZ and WP wrote the manuscript. All authors read and approved the final manuscript.

## Pre-publication history

The pre-publication history for this paper can be accessed here:

http://www.biomedcentral.com/1755-8794/7/11/prepub

## Supplementary Material

Additional file 1: Text S1 Additional description of NMF and the correlation between the K-signature and the expression of APOBEC family genes. Click here for file

Additional file 2: Table S1 Comparison of NMF performance using *r* = 3:7 in each cancer. Click here for file

Additional file 3: Table S2 Mutation burdens (C → T and C → G in the TCX context) versus expression changes of the *APOBEC* family genes. Click here for file

Additional file 4: Table S3 Mutation burdens (C → T and C → G in the TCX context) versus expression changes of the *APOBEC* family genes in samples with ≤ 200 mutations per exome. Click here for file

Additional file 5: Figure S1 *APOBEC3B* gene activity in TCGA cancers. Click here for file

Additional file 6: Figure S2 Comparison of signatures obtained using all SNVs and those obtained excluding non-CpG island (CGI) C → T mutations. Click here for file

Additional file 7: Figure S3 Mutation signature load in melanoma. Click here for file

Additional file 8: Figure S4 The effect of the outlier sample in SQCC. Click here for file
