# Supplementary material for: Patterns and processes of somatic mutations in nine major cancers
Source: BMC Med Genomics. 2014 Feb 19;7:11. doi: 10.1186/1755-8794-7-11 (PMC3942057; doi:10.1186/1755-8794-7-11)
Supplement: Additional file 1: Text S1 — Additional description of NMF and the correlation between the K-signature and the expression of APOBEC family genes. [file 1755-8794-7-11-S1.docx]

**Additional file 1: Text S1**

**Non-negative Matrix Factorization (NMF)**

Without considering DNA strands (e.g., A→G vs. T→C), there are six types of single nucleotide substitutions in the DNA sequence: C:G→A:T, C:G→G:C, C:G→T:A, T:A→A:T, T:A→C:G, and T:A→G:C. For each mutation, we denote the mutation site itself (X→Y) and two nucleotides immediately surrounding the site (A and B) as a unit of trinucleotides A(X→Y)B. For example, for the transversion from C to G in the context of 5’-A and 3’-T, we describe it as 5’-A(C→G)T-3’, or simply A(C→G)T. Accordingly, there are a total of 96 (4×6×4) possible trinucleotides in cancer genomes. The central mutation data structure is a matrix in which each trinucleotide has a corresponding number of occurrences in each sample. This information creates a matrix of 96×*N* (denoted by **M**), where *N* is the number of samples. The algorithm Non-negative Matrix Factorization is then applied to dissect the mutation matrix with a predefined number of processes *r*: **M**_96×_*_N_* = **W**_96×_*_r_*×**H***_r_*_×_*_N_*+ε. The matrix **W**_96×_*_r_* contains *r* columns, each of which represents a mutational signature. We assume that an observed signature in a cancer genome is produced by a major mutational process. The matrix **H***_r_*_×_*_N_* contains *N* columns, where H*_r_*_×_*_j_* = [β*_i_*]^T^ , *i*=1:*r* and *j*=1:*N*. The coefficient β*_i_* represents the load of the *i*^th^ signature on the fraction of mutations in the *j*^th^ sample. For each cancer type, we evaluated the performance of *r* with a value from 3 to 7 and selected the representative *r* value based on the achieved sparseness and cophenetic correlation in that cancer (1,2). Comparisons of the performance of NMF when fitting with r=3-7 in each cancer are provided in Additional file 4: Table S3.

**The K-signature and its correlation with increased expression of APOBEC family genes**

To further understand the biological significance of the observed K-signature, we systematically examined its relevant mutation burden versus the expression change of the APOBEC family genes. Previous studies(3) suggested that the C→T mutations in the TpC dinucleotide context related to the *kataegis* signature might be associated with the AID/APOBEC mediated DNA repair system. In humans, the APOBEC family has 11 members(4). A positive correlation was established in TCGA_BRCA samples between the *APOBEC3B* expression level and the C→T transition burden(3) but not in *APOBEC3G*, another APOBEC family member gene. We defined the mutation burden per exome regarding the K-signature as the sum of T(C→T)X and T(C→G)X mutations, including 8 types of trinucleotides. The overall mutation burden per exome is defined as the sum of all mutations detected in sequences covered by WES. Among the nine cancers we examined, six had gene expression data, all of which were generated by TCGA using RNA sequencing (RNA-seq) (<https://tcga-data.nci.nih.gov/tcga/>). Gene expression was measured using the normalized count values in the RNA-seq data. For each APOBEC family gene, samples were separated into three groups according to its expression level: low (rank between 1-33% of the samples), intermediate (rank between 34-66%), and high (rank between 67-100%).

We first examined *APOBEC3B*. Fig. 3 shows the K-signature related mutation burden versus *APOBEC3B* gene expression in three TCGA cancers in which the K-signature was observed: BRCA, EC, and SQCC. A positive correlation was observed in Fig. 3 between the *APOBEC3B* expression with both the K-signature related mutation burden and the overall somatic mutation burden in BRCA and EC, but not in SQCC. Furthermore, a comparison of the *APOBEC3B* expression levels in all six TCGA cancers revealed that on average, *APOBEC3B* has a generally high expression level in BRCA, EC, and SQCC, a moderate level in OvCa, and a low level in CRC and GBM (Fig. 3). Notably, the K-signature was observed in all three cancers with high *APOBEC3B* gene expression (BRCA, EC, and SQCC) but not in two cancers with a low expression (CRC and GBM). In OvCa, the *APOBEC3B* gene expression is intermediate among the six cancers. The signature #2 of OvCa (Fig. 1) presented high coefficients for C→T and C→G mutations in TCX trinucleotides, though they did not form a recognizable K-signature. Put together, these results strongly support the notion of a positive association between the K-signature related mutation burden and increased *APOBEC3B* gene expression.

In addition to *APOBEC3B*, we further found *APOBEC3A* also had a positive correlation with the K-signature burden. No other genes in the APOBEC family showed a consistent correlation with the K-signature. To reduce potential biases caused by other mutation processes (e.g., mutagen-driven or deficiency in DNA repair genes), we conducted the analysis in all samples and in a subset of samples with ≤ 200 mutations per exome. As shown in Supplementary Table S1 (all samples) and Supplementary Table S2 (samples with ≤ 200 mutations per exome), *APOBEC3A* and *APOBEC3B* consistently showed a positive correlation between their gene expression level and the T(C→T)X and T(C→G)X burden in three cancers: BRCA (with the K-signature), EC (with the K-signature), and OvCa (no recognizable K-signature). However, they did not show the same correlation in SQCC (with the K-signature), CRC (without the K-signature), or GBM (without the K-signature).

Our statistical tests showed that among these cancers, breast tumors had the strongest significant correlation: comparison between the K-signature mutation burden in the samples with high *APOBEC3A* expression versus the samples with low *APOBEC3A* expression had *p* = 9.63×10^-12^, while for *APOBEC3B*, *p* = 8.16×10^-10^ (two-sided Wilcoxon rank sum test). In EC, although the correlation was only marginally significant in all samples (*p*=0.075 for *APOBEC3A* and *p=*0.057 for *APOBEC3B*), it was significant for samples with ≤ 200 mutations per exome (*p*=0.037 for *APOBEC3A* and *p=*0.023 for *APOBEC3B*). This result is likely because that in some EC samples, the mutations were influenced by mutant *POLE* and/or aberrant *MLH1* expression levels (see below). The correlation remains significant in OvCa (*p*=6.90×10^-4^ for *APOBEC3A* and *p*=6.17×10^-4^ for *APOBEC3B*). In SQCC, however, we did not observe a significant difference in the K-signature mutation burden versus either *APOBEC3A* (*p*=0.525) or *APOBEC3B* (*p*=0.602) gene expression groups. A potential reason for this observation is that tobacco exposure inflated the mutations at C nucleotides in lung cancer patients. While we could distinguish the tobacco-related S-signature from the K-signature, it is difficult to determine the proportion of the C→T mutations that is either induced by the increased *APOBEC3A* or *APOBEC3B* expression or shifted by tobacco exposure.

1. Gaujoux, R. and Seoighe, C. (2010) A flexible R package for nonnegative matrix factorization. *BMC Bioinformatics*, **11**, 367.

2. Brunet, J.P., Tamayo, P., Golub, T.R. and Mesirov, J.P. (2004) Metagenes and molecular pattern discovery using matrix factorization. *Proc Natl Acad Sci U S A*, **101**, 4164-4169.

3. Burns, M.B., Lackey, L., Carpenter, M.A., Rathore, A., Land, A.M., Leonard, B., Refsland, E.W., Kotandeniya, D., Tretyakova, N., Nikas, J.B., Yee, D., Temiz, N.A., Donohue, D.E., McDougle, R.M., Brown, W.L., Law, E.K. and Harris, R.S. (2013) APOBEC3B is an enzymatic source of mutation in breast cancer. *Nature*, **494**, 366-370.

4. Prochnow, C., Bransteitter, R. and Chen, X.S. (2009) APOBEC deaminases-mutases with defensive roles for immunity. *Sci. China C Life Sci*, **52**, 893-902.
