# Supplementary material for: Patterns and processes of somatic mutations in nine major cancers
Source: BMC Med Genomics. 2014 Feb 19;7:11. doi: 10.1186/1755-8794-7-11 (PMC3942057; doi:10.1186/1755-8794-7-11)
Supplement: Additional file 3: Table S2 — Mutation burdens (C → T and C → G in the TCX context) versus expression changes of the APOBEC family genes. [file 1755-8794-7-11-S3.docx]

**Additional file 3: Table S2. Mutation burdens (C→T and C→G in the TCX context) versus expression changes of the *APOBEC* family genes.**

|  | TCGA_BRCA | TCGA_CRC | TCGA_EC | TCGA_GBM | TCGA_OvCa | TCGA_SQCC |
| --- | --- | --- | --- | --- | --- | --- |
| # samples | 500 | 221 | 241 | 150 | 163 | 177 |
|  | | | | | | |
| **Absolute gene expression** | | | | | | |
| *APOBEC1* | NA | **0.0139** | NA | NA | NA | NA |
| *APOBEC2* | 0.1565 | 0.2422 | 0.8470 | 0.3630 | 0.8460 | 0.7345 |
| *APOBEC3A* | **9.63×10^-12^** | 0.0296 | **0.0749** | 0.2682 | **6.897×10^-4^** | 0.5253 |
| *APOBEC3B* | **8.16×10^-10^** | 0.3770 | **0.0573** | 0.9448 | **6.170×10^-4^** | 0.6015 |
| *APOBEC3C* | 0.4060 | 0.4461 | 0.9035 | 0.6207 | 0.3197 | 0.1775 |
| *APOBEC3D* | 0.8651 | 0.3400 | 0.2314 | 0.7686 | 0.8727 | 0.6376 |
| *APOBEC3F* | 0.8025 | 0.0046 | 0.3236 | 0.8114 | 0.4230 | 0.4010 |
| *APOBEC3G* | 0.1132 | **0.0225** | 0.1379 | 0.0920 | 0.3830 | 0.6015 |
| *APOBEC3H* | 0.1622 | 0.0971 | 0.1697 | 0.1164 | 0.0635 | 0.7244 |
| *APOBEC4* | NA | NA | **0.0114** | NA | 0.3299 | 0.6147 |
| Both *APOBEC3A* and *APOBEC3B* | **1.83×10^-10^** | 0.1375 | **0.0466** | 0.1378 | **1.655×10^-4^** | 0.2494 |
|  |  |  |  |  |  |  |
| **Gene expression relative to *TBP*** | | | | | | |
| *APOBEC1* | NA | **0.0034** | NA | NA | NA | NA |
| *APOBEC2* | 0.0687 | 0.1810 | 0.8967 | 0.4146 | 0.7532 | 0.3038 |
| *APOBEC3A* | **1.27×10^-11^** | 0.0552 | 0.0760 | 0.2864 | **0.0357** | 0.7528 |
| *APOBEC3B* | **1.45×10^-9^** | 0.2478 | 0.1351 | 0.9201 | **0.0391** | 0.9635 |
| *APOBEC3C* | 0.5478 | 0.4662 | 0.8590 | 0.8572 | 0.1545 | **0.0365** |
| *APOBEC3D* | 0.7176 | 0.1061 | 0.3065 | 0.6157 | 0.8485 | 0.5394 |
| *APOBEC3F* | 0.8479 | **0.0017** | 0.0566 | 0.4676 | 0.5501 | **0.0362** |
| *APOBEC3G* | 0.1364 | **0.0064** | 0.4484 | 0.2364 | 0.7370 | 0.9571 |
| *APOBEC3H* | 0.3513 | 0.0617 | 0.0646 | 0.3893 | 0.0683 | 0.1836 |
| *APOBEC4* | NA | NA | **0.0334** | NA | 0.9165 | 0.6319 |

The number of samples are those with both somatic mutations and gene expression data. *TBP*: a housekeeping gene. p-values < 0.05 are shown in bold.
