# Supplementary material for: Patterns and processes of somatic mutations in nine major cancers
Source: BMC Med Genomics. 2014 Feb 19;7:11. doi: 10.1186/1755-8794-7-11 (PMC3942057; doi:10.1186/1755-8794-7-11)
Supplement: Additional file 4: Table S3 — Mutation burdens (C → T and C → G in the TCX context) versus expression changes of the APOBEC family genes in samples with ≤ 200 mutations per exome. [file 1755-8794-7-11-S4.docx]

**Additional file 4: Table S3. Mutation burdens (C→T and C→G in the TCX context) versus expression changes of the *APOBEC* family genes in samples with ≤ 200 mutations per exome.**

|  | TCGA_BRCA | TCGA_CRC | TCGA_EC | TCGA_GBM | TCGA_OvCa | TCGA_SQCC |
| --- | --- | --- | --- | --- | --- | --- |
| # samples | 481 | 185 | 155 | 149 | 163 | 32 |
|  | | | | | | |
| **Absolute gene expression** | | | | | | |
| *APOBEC1* | NA | 0.6462 | NA | NA | NA | NA |
| *APOBEC2* | 0.1114 | 0.2351 | 0.5482 | 0.3766 | 0.8460 | 0.2635 |
| *APOBEC3A* | **1.20×10^-10^** | 0.6741 | **0.0366** | 0.4161 | **6.897×10^-4^** | 0.0937 |
| *APOBEC3B* | **7.65×10^-10^** | 0.2281 | **0.0227** | 0.7835 | **6.170×10^-4^** | 0.6454 |
| *APOBEC3C* | 0.8998 | 0.5033 | 0.3351 | 0.7536 | 0.3197 | 0.8180 |
| *APOBEC3D* | 0.1872 | 0.5081 | 0.0805 | 0.5827 | 0.8727 | 0.7174 |
| *APOBEC3F* | 0.6689 | 0.1823 | 0.7883 | 0.8726 | 0.4230 | 0.5324 |
| *APOBEC3G* | 0.3864 | 0.9428 | 0.5459 | 0.0729 | 0.3830 | 0.5106 |
| *APOBEC3H* | 0.4661 | 0.7447 | 0.8959 | 0.1945 | 0.0635 | 0.4495 |
| *APOBEC4* | NA | NA | **0.0079** | NA | 0.3299 | 0.0564 |
| Both *APOBEC3A* and *APOBEC3B* | **7.51×10^-10^** | 0.4236 | **0.0084** | 0.1378 | **1.655×10^-4^** | 0.6095 |
|  |  |  |  |  |  |  |
| **Gene expression relative to *TBP*** | | | | | | |
| *APOBEC1* | NA | 0.3465 | NA | NA | NA | NA |
| *APOBEC2* | 0.1161 | 0.3075 | 0.4463 | 0.3082 | 0.7532 | 0.6692 |
| *APOBEC3A* | **3.90×10^-11^** | 0.9836 | **0.0665** | 0.1990 | **0.0357** | 0.2239 |
| *APOBEC3B* | **3.91×10^-10^** | 0.0660 | **0.0217** | 0.8362 | **0.0391** | 0.1481 |
| *APOBEC3C* | 0.7890 | 0.4633 | 0.8667 | 0.9205 | 0.1545 | 0.0525 |
| *APOBEC3D* | 0.2683 | 0.7005 | 0.1775 | 0.6763 | 0.8485 | 0.3929 |
| *APOBEC3F* | 0.4546 | 0.3062 | 0.3405 | 0.3747 | 0.5501 | 1.0000 |
| *APOBEC3G* | 0.3567 | 0.7917 | 0.8510 | 0.3159 | 0.7370 | 0.1883 |
| *APOBEC3H* | 0.7245 | 0.8616 | 0.6749 | 0.5212 | 0.0683 | 0.8694 |
| *APOBEC4* | NA | NA | **0.0073** | NA | 0.9165 | 0.0383 |

The number of samples are those with both somatic mutations and gene expression data. *TBP*: a housekeeping gene. p-values < 0.05 are shown in bold.
