# Supplementary material for: Patterns and processes of somatic mutations in nine major cancers
Source: BMC Med Genomics. 2014 Feb 19;7:11. doi: 10.1186/1755-8794-7-11 (PMC3942057; doi:10.1186/1755-8794-7-11)
Supplement: Additional file 5: Figure S1 — APOBEC3B gene activity in TCGA cancers. [file 1755-8794-7-11-S5.docx]

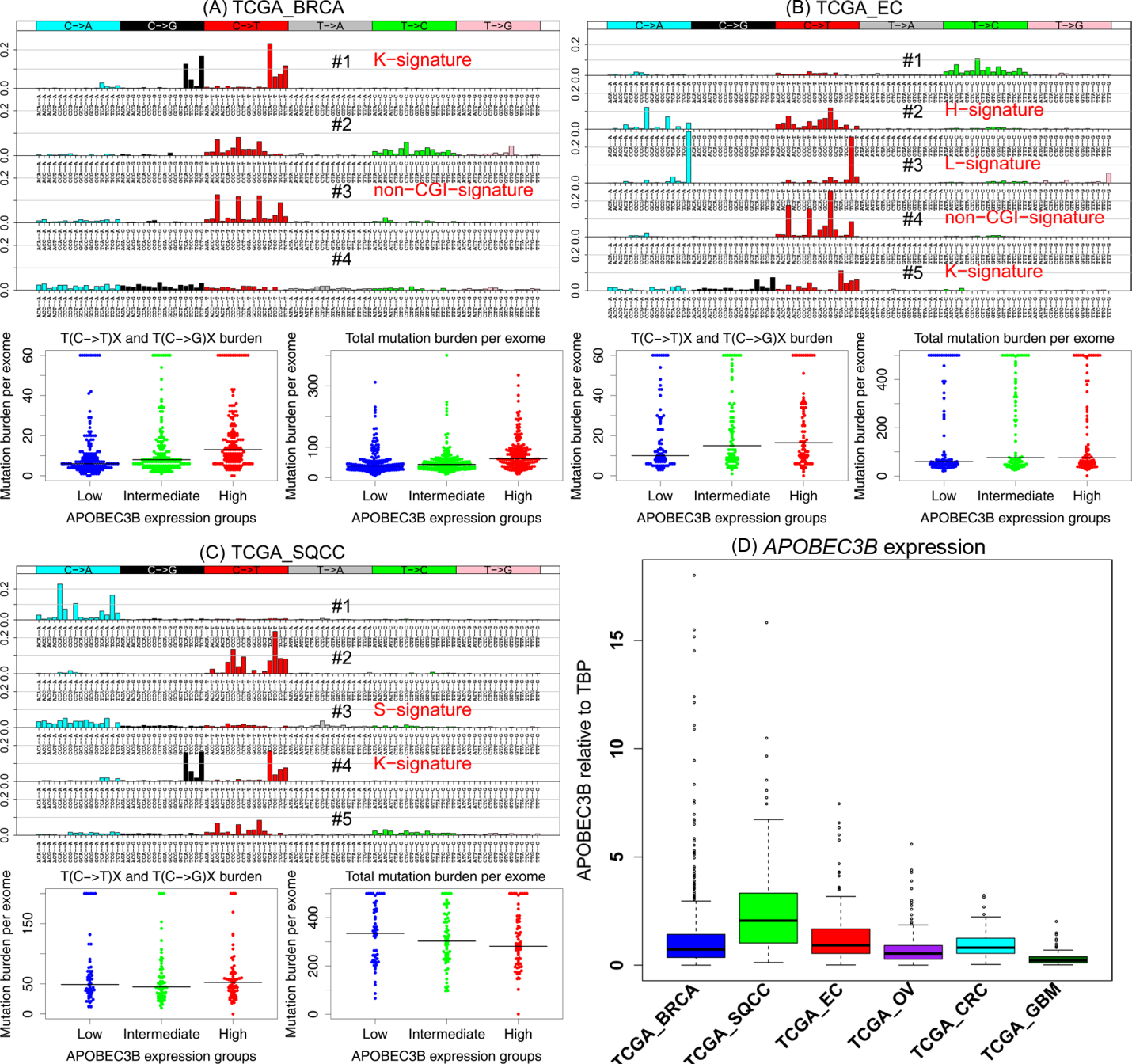


**Additional file 5: Figure S1.** ***APOBEC3B* gene activity in TCGA cancers.**

Mutation burdens versus *APOBEC3B* gene expression in TCGA_BRCA (A), TCGA_EC (B), and TCGA_SQCC (C) samples are displayed respectively. The mutation burden measured by the K-signature (the sum of C→T and C→G mutations in TCX) and the total mutation burden were plotted using three groups of samples with low (rank 1-33%), intermediate (rank 34-66%), and high (rank 67-100%) expression of *APOBEC3B* gene. The overall *APOBEC3B* gene expression in all 6 TCGA cancers was plotted in (D). Note that we use a relative expression of the *APOBEC3B* gene by comparing it to the expression of a housekeeping gene *TBP* to provide a fair comparison across multiple cancers (D).
