# Supplementary material for: Patterns and processes of somatic mutations in nine major cancers
Source: BMC Med Genomics. 2014 Feb 19;7:11. doi: 10.1186/1755-8794-7-11 (PMC3942057; doi:10.1186/1755-8794-7-11)
Supplement: Additional file 6: Figure S2 — Comparison of signatures obtained using all SNVs and those obtained excluding non-CpG island (CGI) C → T mutations. [file 1755-8794-7-11-S6.docx]

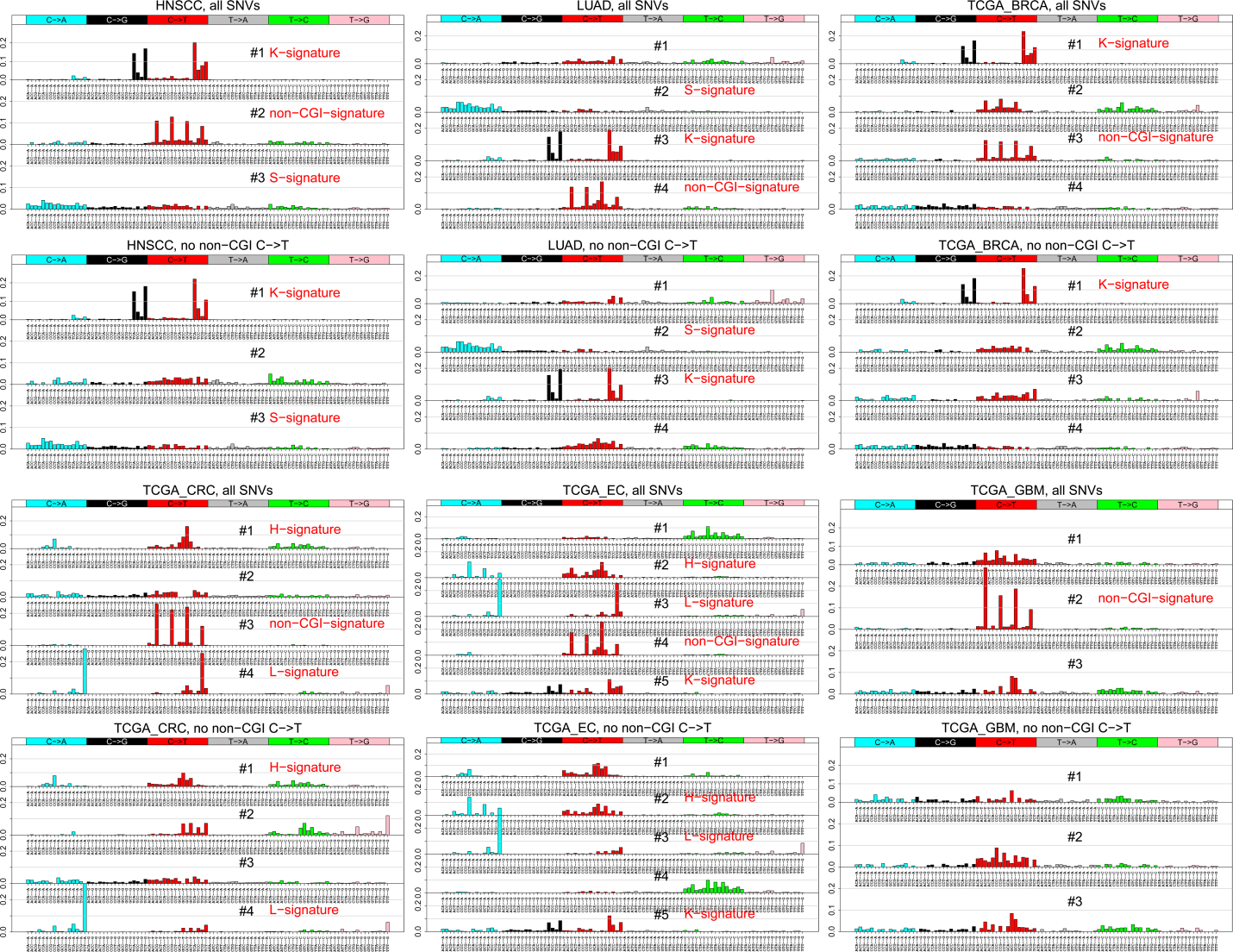


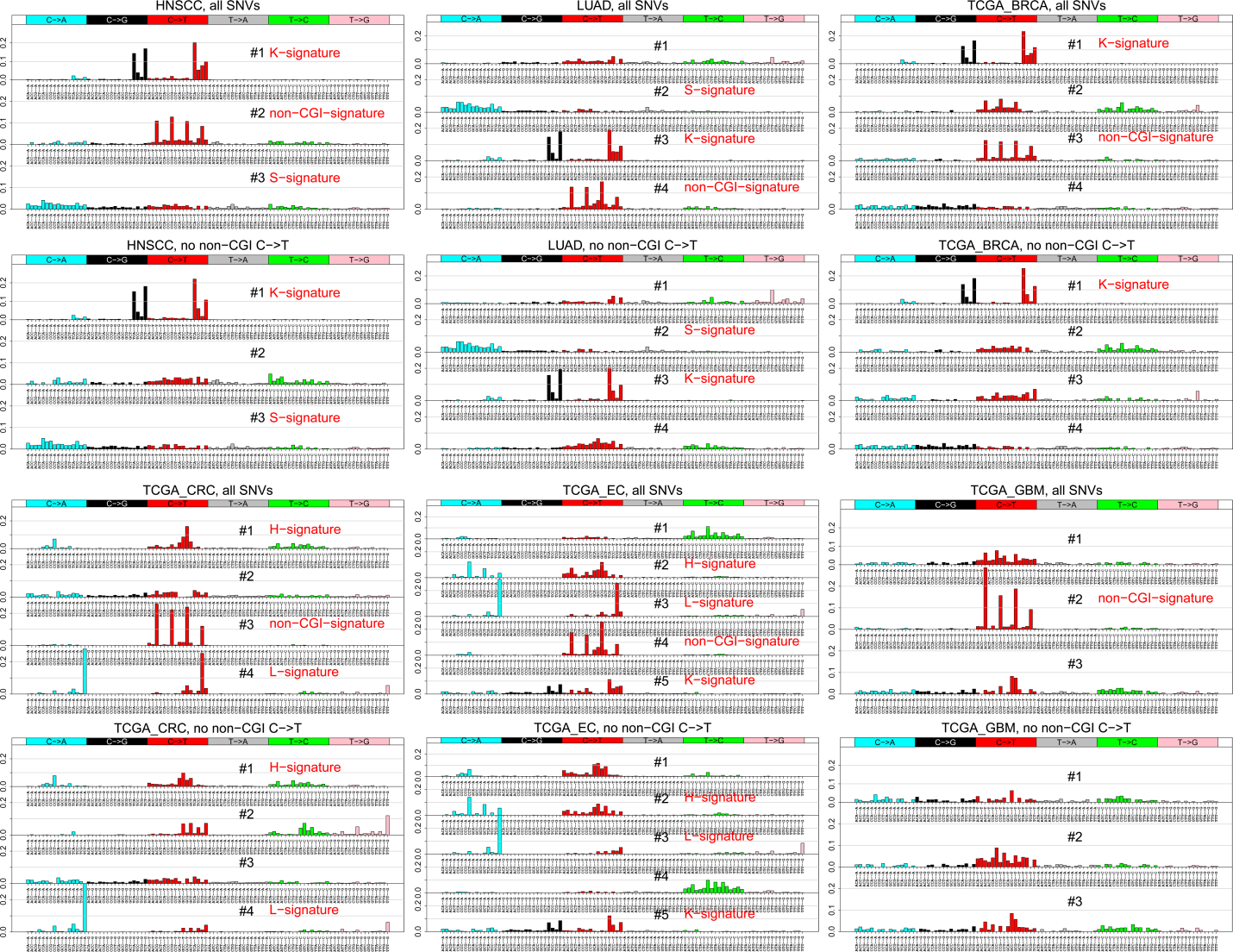


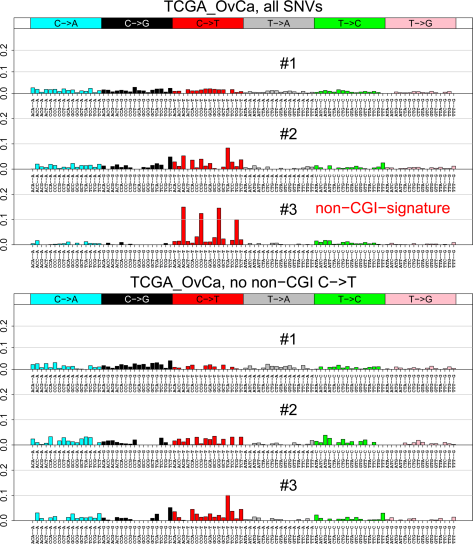


**Additional file 6: Figure S2.** **Comparison of signatures obtained using all SNVs and those obtained excluding non-CpG island (CGI) C→T mutations.**

In all 7 cancers, the non-CGI signature disappeared when excluding those in non-CGI regions.
