# Supplementary material for: Patterns and processes of somatic mutations in nine major cancers
Source: BMC Med Genomics. 2014 Feb 19;7:11. doi: 10.1186/1755-8794-7-11 (PMC3942057; doi:10.1186/1755-8794-7-11)
Supplement: Additional file 7: Figure S3 — Mutation signature load in melanoma. [file 1755-8794-7-11-S7.docx]

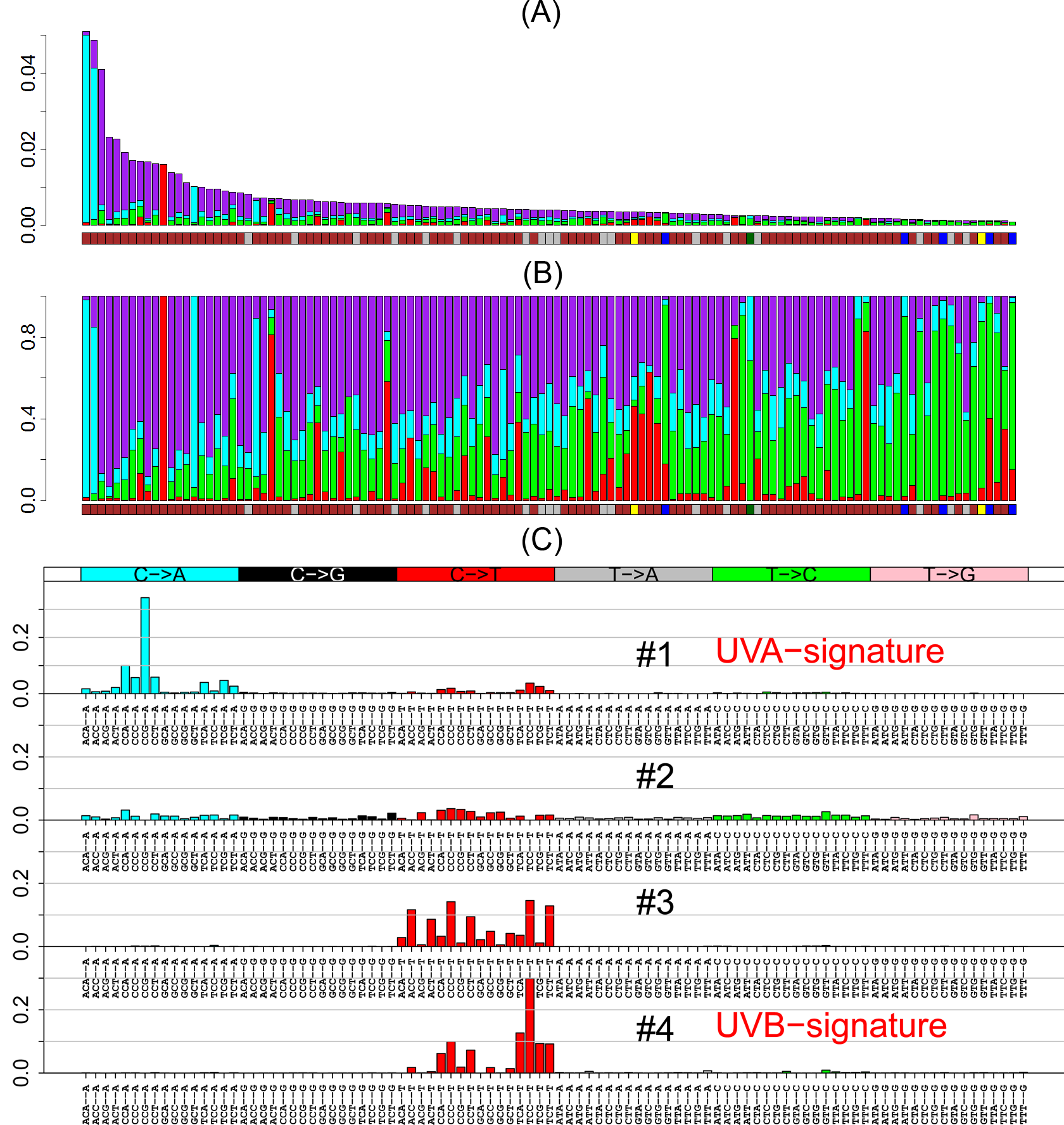


**Additional file 7: Figure S3.** **Mutation signature load in melanoma.**

Contribution of the signatures to each melanoma sample is represented by the actual coefficients (A) and the relative coefficients (B). A vertical bar with 4 colors represents a melanoma sample. The 4 colors represent the 4 mutation signatures detected in melanoma in the same order as in (C): red for signature #1, green for signature #2, cyan for signature #3, and purple for signature #4. The horizontal bars in the bottom of (A) and (B) represent sample types: melanoma of skin is in brown; mucosal in yellow; uveal in dark green, and acral in blue. Acral and mucosal melanomas tend to have low load of UVA-/UVB-signatures (A).
