# Supplementary material for: Patterns and processes of somatic mutations in nine major cancers
Source: BMC Med Genomics. 2014 Feb 19;7:11. doi: 10.1186/1755-8794-7-11 (PMC3942057; doi:10.1186/1755-8794-7-11)
Supplement: Additional file 8: Figure S4 — The effect of the outlier sample in SQCC. [file 1755-8794-7-11-S8.docx]

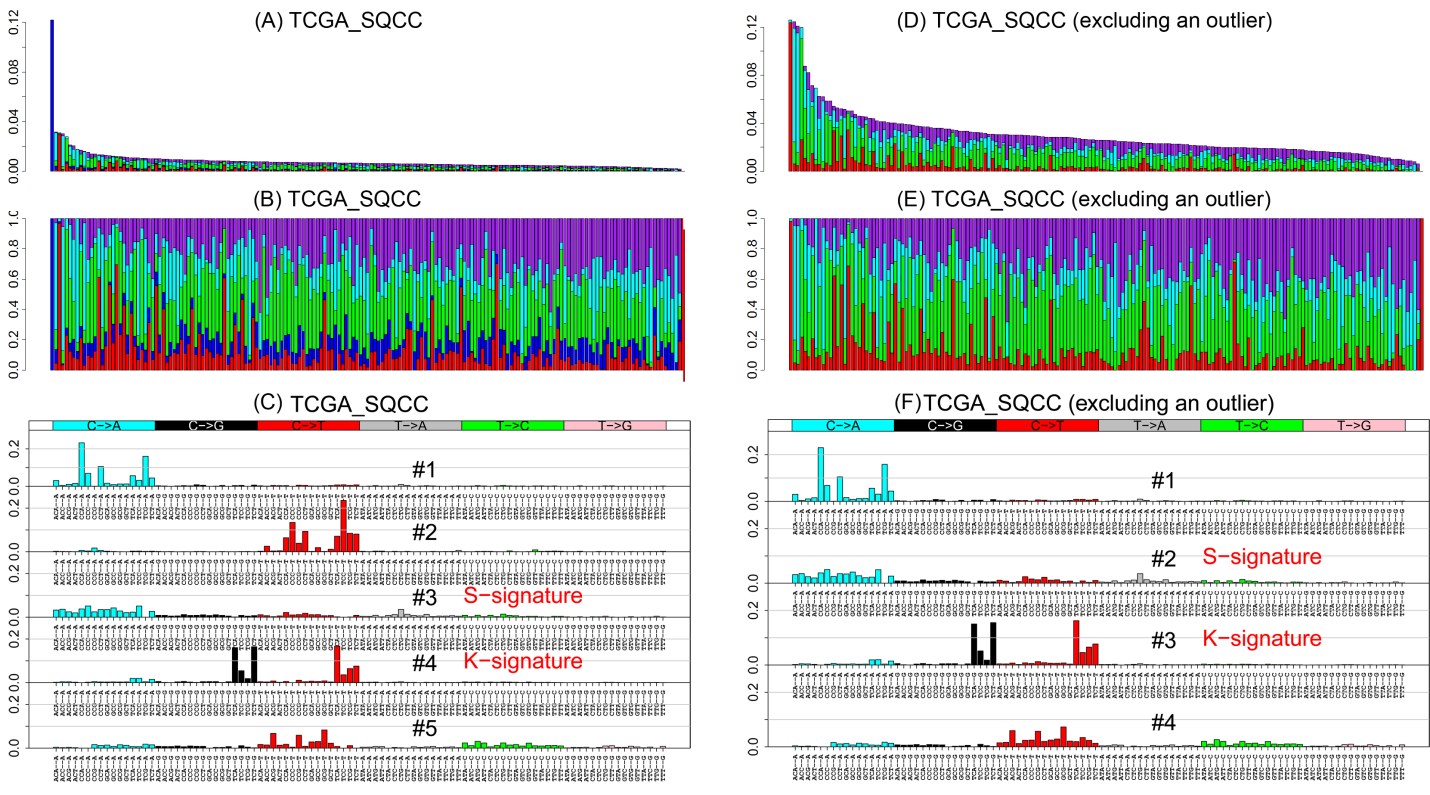


**Additional file 8: Figure S4.** **The effect of the outlier sample in SQCC.**

The left panel (A, B, and C) represent mutations for all SQCC samples and the right panel (D, E, and F) for SQCC samples excluding one outlier sample (i.e., the first sample in (A) with abnormally high load of mutation signatures). Contribution of the signatures to each SQCC sample is represented by the actual coefficients (A and D) and the relative coefficients (B and E). A vertical bar in (A), (B), (D), and (E) with represents a SQCC sample. The colors represent the mutation signatures as shown in (C) and (F), respectively. In the scenario of (A-C), 5 signatures are represented: red for signature #1, blue for signature #2, green for signature #3, cyan for signature #4, and purple for signature #5. In the scenario of (D-F), 4 signatures are represented: red for signature #1, green for signature #2, cyan for signature #3, and purple for signature #4. One signature in the left scenario (#2 in C and blue bars in A and B) disappeared after excluding the outlier sample (F).
